# Spinal Cord Compression Syndrome Revealing Thoracic Ewing Sarcoma in a Young Adult: A Case Report

**DOI:** 10.7759/cureus.117444

**Published:** 2026-10-04

**Authors:** Kamal Bouchkara, Karim BAAYOUD, Naji KADIRI ALAOUI, Zakariae Benyaich, Mohamed LMEJJATI

**Affiliations:** 1 Neurosurgery, Faculty of Medicine and Pharmacy Ibn Zohr University, Mohammed VI University Hospital, Agadir, MAR; 2 Neurosurgery, Faculty of Medicine and Pharmacy, Ibn Zohr University, Mohammed VI University Hospital, Agadir, MAR; 3 Neurosurgery, Mohammed VI University Hospital, Faculty of Medicine and Pharmacy, IBN Zohr University, Agadir, MAR

**Keywords:** ewing sarcoma, spinal cord compression, spinal neoplasms, thoracic spine, young adult

## Abstract

Ewing sarcoma is a primary malignant bone tumor that predominantly affects children and adolescents. It most commonly arises in the pelvis, chest wall, and long bones of the extremities. However, involvement of the thoracic spine is exceptionally rare. We report the case of a 20-year-old patient who presented with thoracic spinal cord compression syndrome as the initial manifestation of thoracic Ewing sarcoma, with nonspecific radiological findings. The diagnosis was confirmed by histopathological examination of the surgical resection specimen.

## Introduction

Ewing sarcoma (ES) is a highly malignant primary bone tumor that predominantly affects children and adolescents [1]. It primarily affects the metaphyses of the long bones, with spinal involvement being much rarer [2]. Spinal forms are divided into two groups: sacral and non-sacral. Moreover, this tumor is exceptionally rare in young adults, and even rarer in the latter. The clinical presentation and radiological findings are nonspecific.

The management of ES, regardless of their location, involves diagnostic biopsy followed by chemotherapy, then surgical resection and/or local radiation therapy, along with another course of induction chemotherapy [3]. Regarding primary spinal Ewing sarcomas, there is no consensus on first-line therapy [4]. However, the prognosis remains guarded in metastatic cases. [4]

## Case presentation

We report the case of a 20-year-old male with no significant past medical history who presented to the emergency department with symptoms of spinal cord compression that had been present for 10 days, characterized by heaviness in both lower limbs and sphincter dysfunction manifesting as urinary incontinence, with no associated signs of infection and no change in his general condition. Clinical examination revealed flaccid paraplegia with complete motor deficit (0/5), no signs of pyramidal tract involvement, a sensory level at T10, and a left paravertebral mass. The remainder of the physical examination was unremarkable.

Magnetic resonance imaging (MRI) revealed an extensive intraspinal lesion extending from D10 to D12, appearing hyperintense on T1-weighted images and with mild T2 hyperintensity, which enhanced after gadolinium injection and extended into the left paravertebral soft tissues. (Figures 1, 2) Thoraco-abdominal-pelvic CT did not identify any other sites of involvement. The patient underwent a laminectomy of the D10 through D12 vertebrae with wide excision of the intraspinal and left paravertebral processes, with no intraoperative complications. The postoperative course was uneventful, with partial recovery of the deficit to 2/5. 

**Figure 1:**
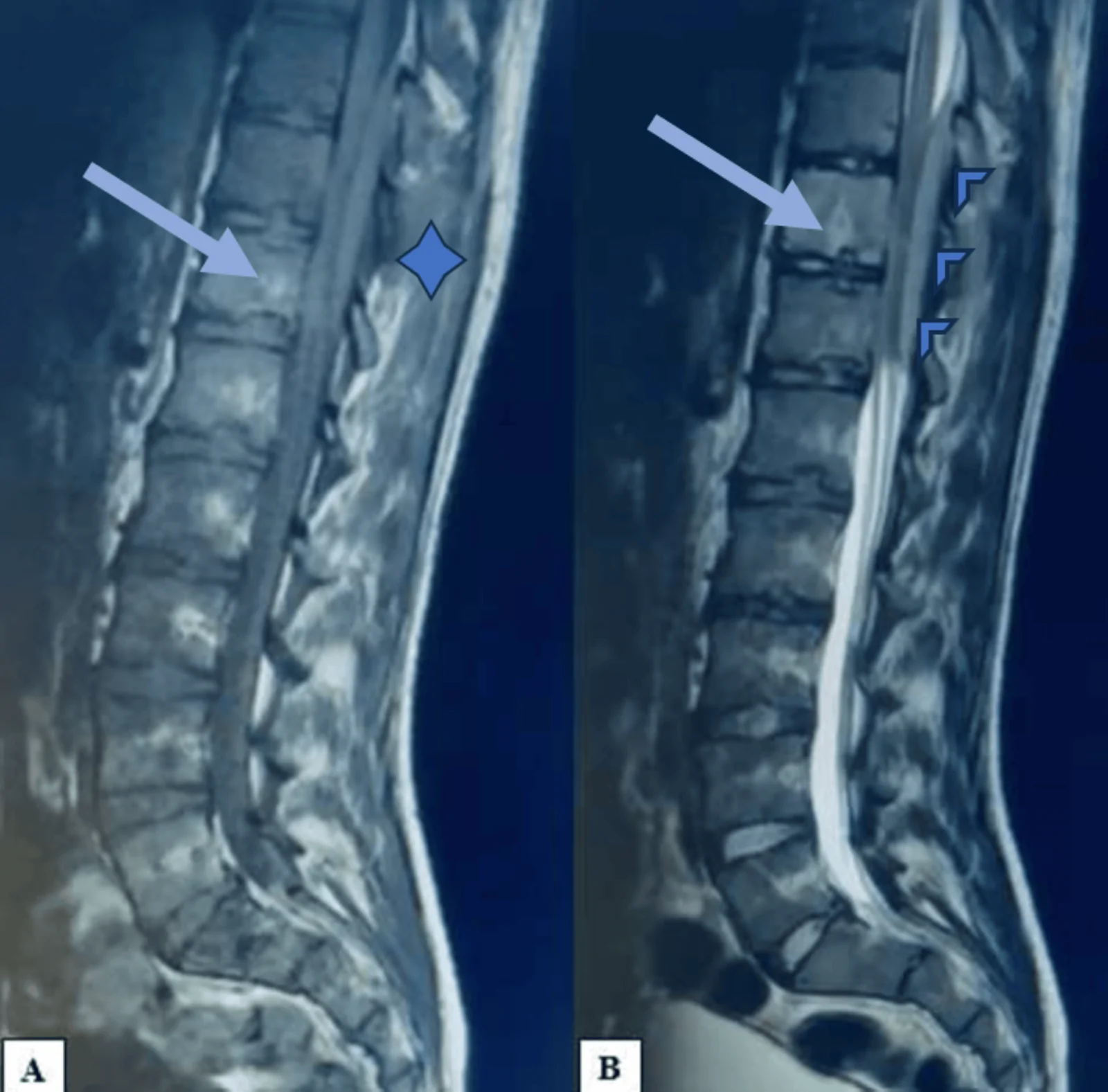
Preoperative spinal MRI in sagittal sections: (A) T1-weighted after gadolinium injection; (B) T2-weighted MRI revealed a lesion with T1 hyperintensity and mild T2 hyperintensity that enhanced after gadolinium injection, involving the D10, D11, and D12 vertebrae (arrow), extending into the posterior soft tissues (star), and causing compressive posterior epiduritis (arrowhead).

**Figure 2:**
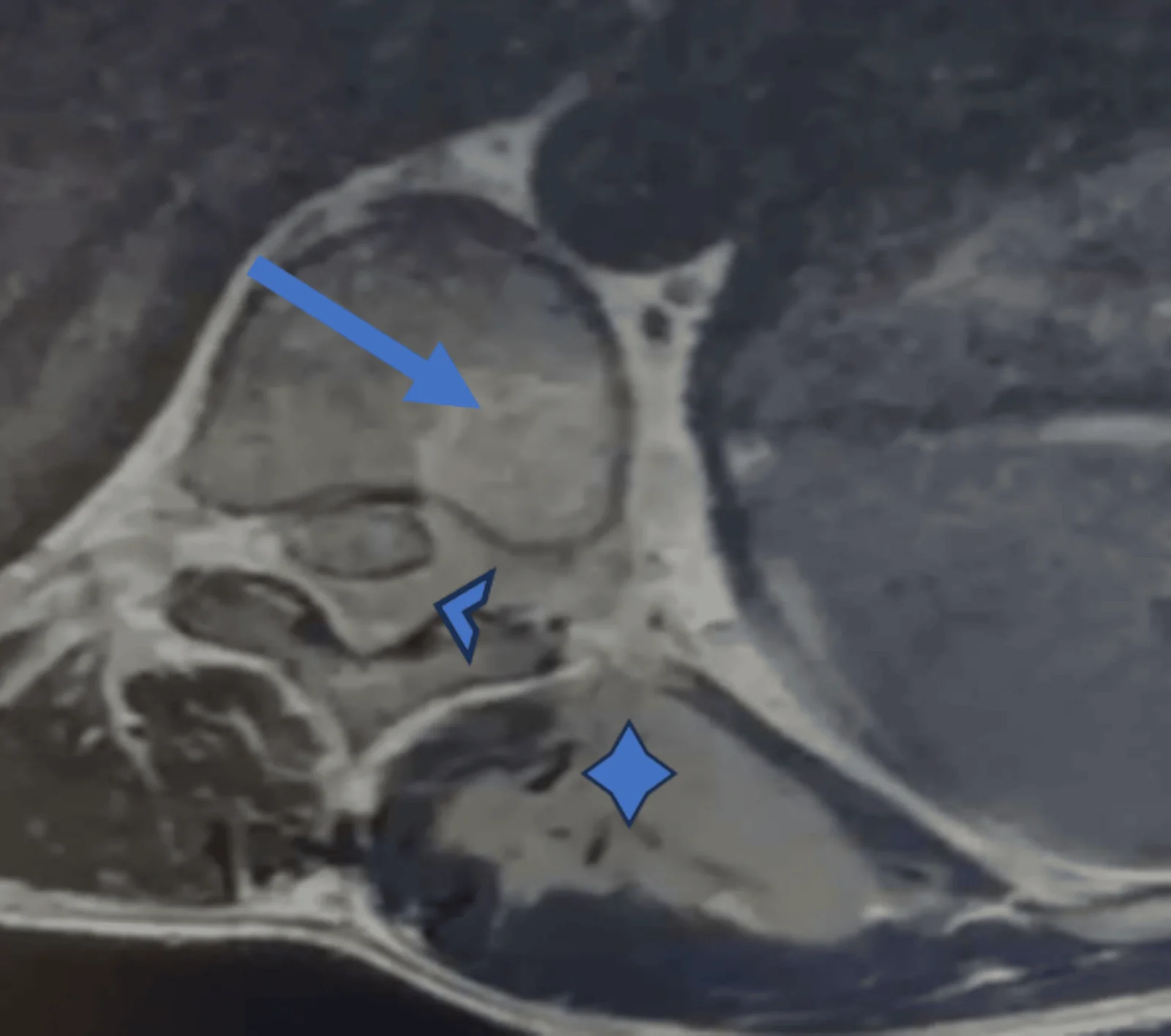
T1-weighted axial MRI of the spinal cord after gadolinium injection Magnetic resonance imaging (MRI) demonstrating involvement of the vertebral body (arrow), posterior epidural extension (arrowhead), and extension into the left paravertebral muscles (star) by Ewing sarcoma.

The pathological examination confirmed the diagnosis of Ewing’s sarcoma (Figure 3). The patient was referred to the oncology department, where he received chemotherapy according to the VAC protocol and stereotactic radiation therapy at a dose of 30 Gy, administered in 10 fractions. He also underwent physical therapy during his hospitalization and subsequently on an outpatient basis, alongside his oncological treatment.

**Figure 3:**
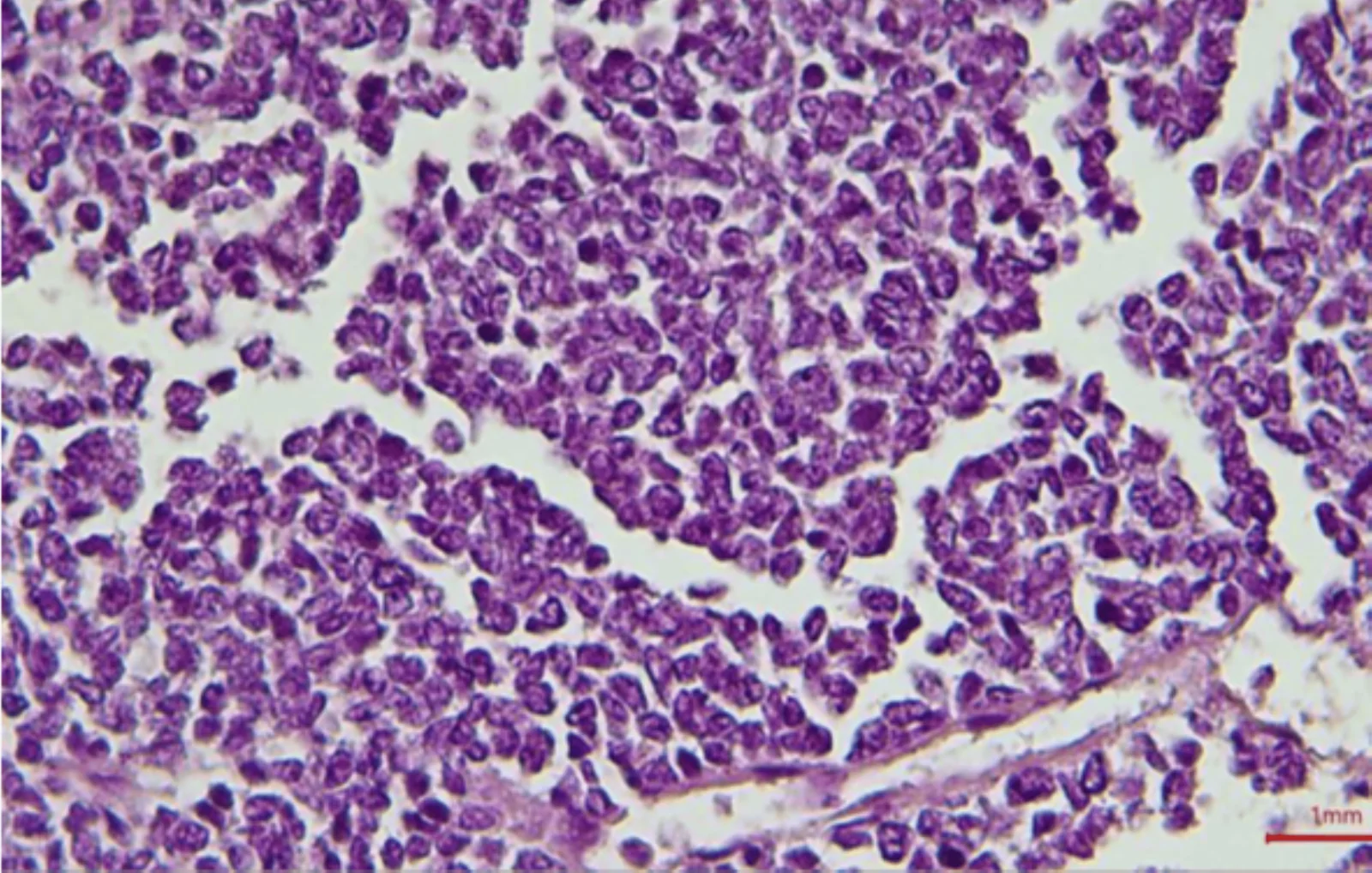
Histopathological image under light microscopy Malignant tumor consisting of undifferentiated round cells.

Six months later, the patient developed headaches and exophthalmos; A brain MRI was performed for these symptoms and revealed an orbital lesion (Figure 4). The patient’s case was reviewed by the oncology team to determine whether another course of chemotherapy was warranted.

**Figure 4:**
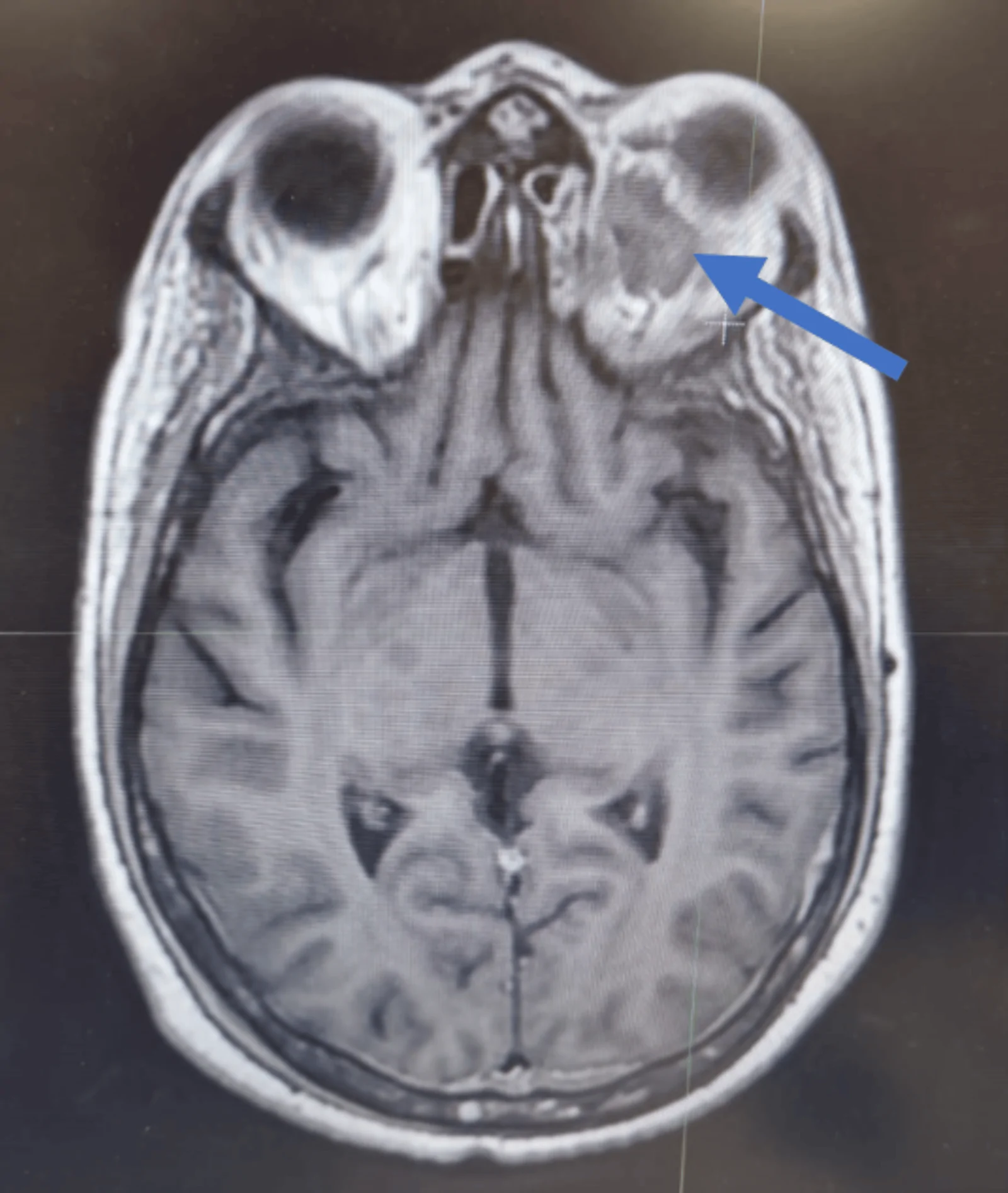
Axial T1-weighted brain MRI after gadolinium injection An extra-conical tissue expansion (arrow) of the left eyeball causing grade 3 exophthalmos associated with meningeal thickening.

## Discussion

Ewing sarcomas (ES) are part of a family of tumors that share the same histological and immunohistochemical characteristics, as well as the presence of the t(11;22) chromosomal translocation [2]. Spinal involvement may be primary or secondary. However, non-sacral spinal involvement is much rarer, estimated to account for between 3.5 and 14.9% of all bone involvement [5]. Neurological manifestations are not uncommon, as emphasized by Hesla et al. [6]. Imaging findings are nonspecific, with a predominance of osteolytic lesions extending into the surrounding soft tissues. This may pose a diagnostic challenge, particularly in differentiating Ewing sarcoma from other neoplastic lesions such as rhabdomyosarcoma, lymphoma, and Langerhans cell histiocytosis. Spinal magnetic resonance imaging (MRI) remains the gold standard for assessing the extent of the lesion [5]. In cases of neurological deficits, the latest guidelines from the European Society for Pediatric Oncology (SIOPE) recommend surgical decompression before starting chemotherapy [7]. However, it is important to note the risk of dissemination associated with surgery [6]. Radiotherapy plays an important role in consolidating the therapeutic response achieved with surgical resection and chemotherapy. The prognosis of metastatic Ewing sarcoma remains poor, whereas patients with localized disease have an estimated overall survival rate of 65-80% [4].

## Conclusions

Thoracic spine involvement is a rare presentation of Ewing sarcoma. Its clinical presentation and imaging findings are often nonspecific, although neurological deficits are not uncommon. In patients presenting with neurological impairment, prompt surgical decompression with the most oncologically appropriate resection should be considered as part of a multimodal treatment strategy combining chemotherapy and radiotherapy. Despite these therapeutic advances, the prognosis remains poor for metastatic forms of the disease.
